# *RUNX3* methylation and anti-tumor immunity

**DOI:** 10.18632/oncoscience.242

**Published:** 2015-09-12

**Authors:** Trevelyan R. Menheniott, Louise M. Judd, Andrew S. Giraud

**Affiliations:** Murdoch Children's Research Institute, Department of Paediatrics, University of Melbourne, Melbourne, Victoria, Australia

**Keywords:** cancer, DNA methylation, epigenetics, RUNX3, anti-tumor immunity

Altered DNA methylation is a hallmark of cancer genomes and has been advocated as both a marker and mechanism of the malignant phenotype. In the conventional view, such alterations include global DNA hypomethylation, linked to aberrant expression of oncogenic driver genes and more localised *de novo* hypermethylation at ‘CpG-island’ promoters, argued to cause inappropriate silencing of tumor suppressor genes. Although paradigms like these have been unquestionably influential, deregulation of the epigenome is not the only source of altered DNA methylation in tumors. Cell lineage and tissue composition have recently emerged as additional determinants of cancer-specific methylation patterns [1, 2].

During differentiation, cell lineages acquire stable and heritable gene expression programs associated with characteristic epigenetic marks, including DNA methylation. Lineage-specific DNA methylation profiles can therefore serve as ‘epigenetic fingerprints’ of cellular identity, suggesting their application as diagnostic biomarkers for diverse cell types. Screening for subliminal lineage signatures concealed within ‘DNA methylomes’ may allow simultaneous detection and enumeration of multiple cell types in complex tissues [3], including cancers [2]. Indeed, methylation signatures reflecting the presence of key prognostic indicators, such as T lymphocytes, have already shown immense promise in predicting clinical outcome of cancers and response to therapy [2].

Gastric cancer (GC), which has one of the highest malignancy-related mortality rates globally, arises through stepwise, inflammation-dependent transformation of gastric epithelial cells. Improved detection of the premalignant inflammatory stages of GC may facilitate earlier intervention and better outcomes [4]. In a recent study by Kurklu *et al*. [5] we described an aberrant hypomethylation signature at the *RUNX3* locus, associated with GC progression, specifically within an upstream alternative promoter (*RUNX3* P1). We found that, based on the degree of *RUNX3* P1 hypomethylation, premalignant gastric epithelial lesions and tumors could be segregated from disease-free tissues. This novel signature may therefore be diagnostic for premalignant inflammation in individuals who are, by definition, at risk of developing GC.

**Figure 1:**
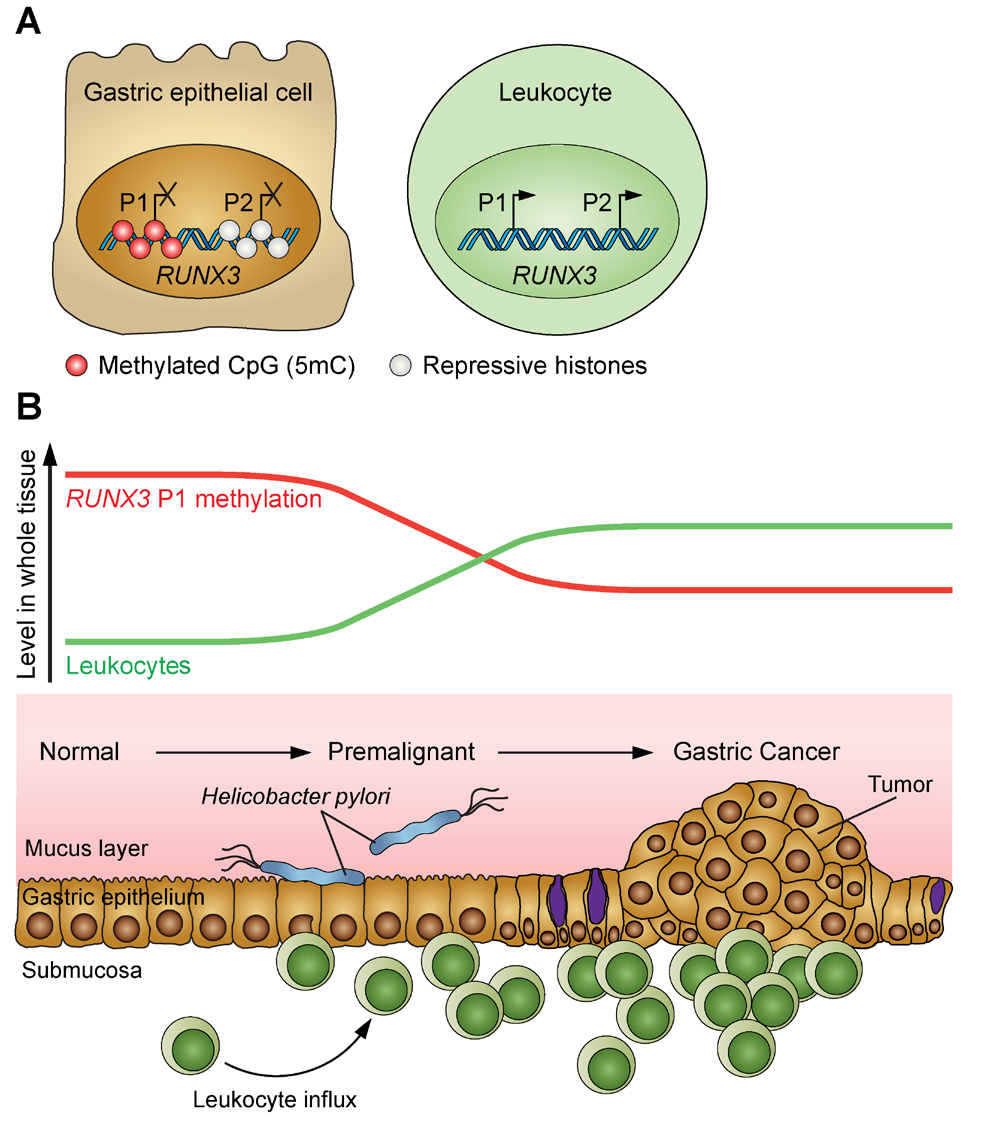
*RUNX3* P1 methylation levels reflect leukocyte infiltration during GC progression **A.** *RUNX3* P1 shows differential DNA methylation (5′methyl cytosine; 5 mC) between gastric epithelial cell and leukocytes. *RUNX3* P2, like most CpG island promoters in normal cells, is controlled by histone modification, not DNA methylation. **B.** Normal (disease-free) gastric epithelium lacks inflammation. Premalignant inflammation (triggered by *Helicobacter pylori* infection) and subsequent tumor growth is characterised by progressive leukocyte infiltration. This change in cell type composition is signified by a corresponding decrease in *RUNX3* P1 methylation levels.

Importantly, our analysis utilised whole tissues/tumors comprised primarily by cancer epithelial cells, but also by non-neoplastic cell types including leukocytes. It had not escaped our attention that *RUNX3*, a key regulator of haematopoiesis, is highly expressed in several immune cell lineages [6]. We therefore hypothesised that enrichment of *RUNX3* P1 hypomethylated alleles in these tissues might reflect leukocyte recruitment to the premalignant epithelium and/or tumor microenvironment. DNA methylation analysis of epithelial and immune cell lineages independently isolated directly from gastric tumors confirmed this expectation; *RUNX3* P1 was hypermethylated and repressed in the epithelial cells but, critically, lacked methylation and was transcriptionally active in all the isolated immune cell types tested. Therefore, *RUNX3* P1 hypomethylation, as a ‘pan’-marker of haematopoietic lineages, can distinguish progressive changes in cellular composition that accompany GC pathogenesis, namely those due to leukocyte infiltration (Figure 1).

A key question not addressed in our study is whether *RUNX3* P1 methylation, as a surrogate of leukocyte influx, might classify gastric tumors into useful prognostic categories? As a case in point, lymphocyte-rich GC generally has a better prognosis than GC lacking significant lymphocytic infiltrate [7]. Intra-tumoral lymphocytes often display cytolytic properties positively linked to survival, hence defining specific methylation signatures of cancers that elicit this type of anti-tumor response would be of high translational priority. The value of such an approach has been shown to great effect in breast cancer. Dedeurwaerder *et al*. described a multi-gene methylation signature that could not only discern the degree of tumor infiltration by T lymphocytes, but was also strongly prognostic for relapse-free survival [2]. They concluded that their signature was likely indicative of an anti-tumor T cell response. While the jury is still out, *RUNX3* P1 methylation has links with key mediators of anti-tumor responses that warrant exploration of its use in similar context. For example, *RUNX3* P1 hypomethylation is a feature of cytolytic CD8+ T lymphocytes and natural killer cells (NK) [5], correlating with both the high *RUNX3* mRNA expression of these two cell types and genetic requirement of *RUNX3* for their intrinsic ‘tumor killing’ functions [6]. Nevertheless, we caution against over interpretation of these observations, as *RUNX3* P1 hypomethylation also marks other immune lineages that can elicit both pro- and anti-tumor effects. Ultimately, whether *RUNX3* P1 methylation can reveal the specific polarity of cancer immune responses, used alone or in combination, awaits formal validation in appropriately powered clinical studies.

We are not the first to study *RUNX3* methylation as a cancer biomarker. Aberrant hypermethylation of the canonical *RUNX3* CpG island promoter (*RUNX3* P2) hitherto proposed [8], but since refuted as a driver of GC has been avidly pursued as a biomarker, albeit with limited success (reviewed in [6]). However, our analysis of *RUNX3* P1 is unique in the field, providing fresh perspectives on cell lineage as a modifier of cancer methylomes and further highlighting its potential for translation in GC as well as other malignancies in which leukocyte infiltration has diagnostic and prognostic significance.
